# Transcranial Pulse Stimulation (TPS®) as a method for treating the central nervous system of patients with Alzheimer’s disease

**DOI:** 10.1192/j.eurpsy.2023.1983

**Published:** 2023-07-19

**Authors:** M. Ziegenbein, D. Clark, V. Rößner-Ruff

**Affiliations:** 1 Psychiatry and Psychotherapy; 2 Old Age Psychitary; 3 Psychiatry Research, Klinikum Wahrendorff, Sehnde, Germany

## Abstract

**Introduction:**

Dementia - one of the most common diseases in old age - is often only diagnosed at a late stage. Therefore patients with dementia have often a 1.4 to 3.6 times greater risk of treatment as an inpatient. Consequently it is highly relevant within the caring system to identify and treat the onset of dementia at the earliest possible opportunity.

**Objectives:**

Part of a new treatment center, a psychiatric clinic in the Hanover area (Wahrendorff ) has concentrated on treating patients with a mild or moderate form of Alzheimer’s disease as early as possible on an outpatient basis. The method of transcranial pulse stimulation (TPS®) is used. Acoustic pulses generated outside the body are introduced specifically into the brain regions requiring treatment. The aim being the release of growth factors and an improvement in cerebral blood flow, as a means to maintaining and promoting cognitive performance for as long as possible. The poster contribution shows reports from clinicians, patients and relatives, using TPS®. The development of cognitive performance in the course of treatment is also considered.

**Methods:**

The data collection for the quantitative study design will take place at the clinic in the period from 06/2021 to 10/2022 (N planned = 60). Cognitive performance is recorded using the Montreal Cognitive Assessment (MoCA test) and the experience reports via interview.

**Results:**

Results of repeated measurement and analysis of the variance in terms of cognitive performance (MoCA test, baseline and follow-up measures) are presented. Field reports are considered and the suitability of TPS® as a method for treating the symptoms of dementia in Alzheimer’s disease is discussed in the form of a best-practice example.

**Conclusions:**

Field reports are considered and the suitability of TPS® as a method for treating the symptoms of dementia in Alzheimer’s disease is discussed in the form of a best-practice example.

**Disclosure of Interest:**

None Declared

